# Primary extraskeletal mesenchymal chondrosarcoma of the colon: a rare case report

**DOI:** 10.1093/gastro/goag075

**Published:** 2026-07-13

**Authors:** Hongjin Liu, Junling Zhang, Xin Wang

**Affiliations:** Department of Gastrointestinal Surgery, Peking University First Hospital, Beijing 100034, P. R. China; Department of Gastrointestinal Surgery, Peking University First Hospital, Beijing 100034, P. R. China; Department of Gastrointestinal Surgery, Peking University First Hospital, Beijing 100034, P. R. China

## Introduction

Extraskeletal mesenchymal chondrosarcoma (EMC) is an exceptionally rare and highly aggressive subtype of chondrosarcoma, accounting for ∼1% of all chondrosarcoma cases. Unlike conventional osseous chondrosarcomas, EMC arises from primitive multipotent mesenchymal cells in extraosseous soft tissues and most often affects adolescents and young adults, typically involving the orbit, central nervous system, craniofacial soft tissues, or deep soft tissues of the lower extremities [1]. Primary visceral involvement is exceedingly uncommon. A retroperitoneal EMC involving the colon was reported in 2021 [2]. Herein, we describe an exceptional case of EMC arising primarily from the colonic wall.

## Case report

A 74-year-old woman presented to the Emergency Department with chills, high fever, nausea, vomiting, and decreased urine and stool output, without abdominal pain or hematochezia. Her medical history included hypertension and penicillin allergy. Physical examination revealed a firm, hard mass below the xiphoid process with unclear margins and mild tenderness to palpation. Empiric intravenous moxifloxacin hydrochloride 0.4 g once daily produced only slight improvement.

**Figure 1:**
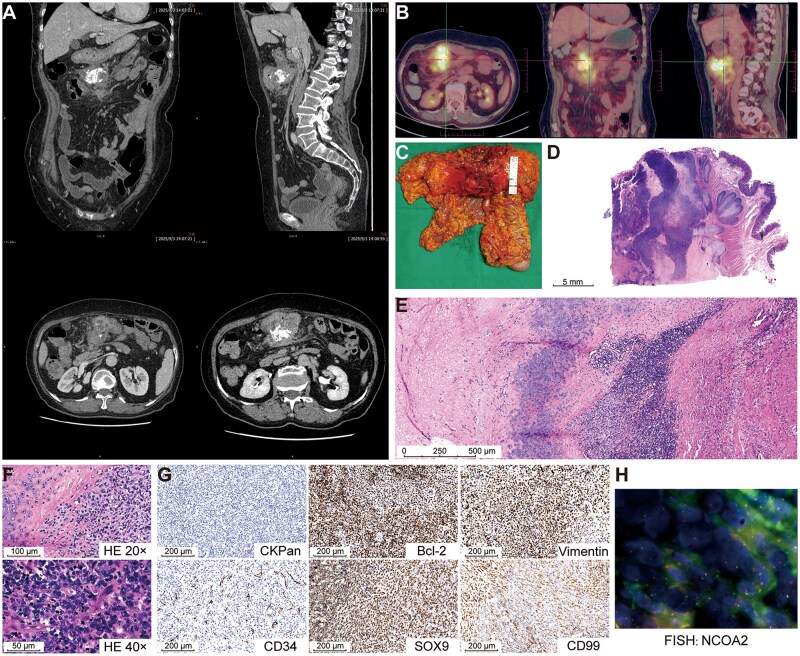
Clinical, radiological, and pathological findings of primary colonic extraskeletal mesenchymal chondrosarcoma. (A) Abdominal contrast-enhanced computed tomography reveals a 7.1 × 5.5 × 5.1 cm mass in the transverse colon with dense heterogeneous calcifications and irregular enhancement, invading adjacent structures. (B) Positron emission tomography-computed tomography imaging demonstrates intense focal hypermetabolism within the tumor (maximum standardized uptake value, 24.8) and adjacent peritumoral nodules. (C) Macroscopic view of the resected specimen, showing a firm nodular intramural mass with mucosal ulceration. (D–F) Hematoxylin and Eosin (HE) staining illustrates the classic biphasic pattern, with highly cellular sheets of undifferentiated small blue round mesenchymal cells interspersed with islands of hyaline cartilage and bone-like matrix. (G) Immunohistochemistry shows positivity for Vimentin, Bcl-2, SOX9, CD99, and CD34, and negativity for CKPan. (H) Fluorescence *in situ* hybridization (FISH) confirms *NCOA2* gene rearrangement in neoplastic cells.

Abdominal contrast-enhanced computed tomography showed marked thickening of the middle transverse colon and a 7.1 cm × 5.5 cm × 5.1 cm mixed-density soft-tissue mass with dense irregular calcifications and heterogeneous enhancement (Fig. 1A). The lesion appeared locally aggressive, with suspected invasion of the gastrocolic ligament and indistinct planes with the posterior gastric wall and pancreatic capsule. Peritumoral inflammatory exudation, encapsulated fluid, and omental thickening with suspicious nodules were present. Positron emission tomography-computed tomography demonstrated intense uptake in the tumor and adjacent exudative nodules (maximum standardized uptake values of 24.8 and 8.8, respectively) (Fig. 1B). A complicated locally advanced colon cancer or severe inflammatory granulomatous disease was considered.

Because of impending obstruction and sepsis, laparoscopic exploration was performed. The transverse colonic mass was densely adherent to the posterior gastric wall, pancreatic capsule, and small-intestinal mesentery, with local abscess formation. En bloc extended right hemicolectomy, partial gastrectomy, and partial resection of the pancreatic capsule were performed. Both intestinal margins were negative. Gross examination showed a firm nodular intramural mass with mucosal ulceration (Fig. 1C). Microscopically, the tumor showed a classic biphasic architecture, consisting of cellular nodules and sheets of small undifferentiated round-to-oval cells with scant cytoplasm, granular hyperchromatic nuclei, frequent mitotic activity (11 mitoses per high-power field), and a hemangiopericytoma-like vascular pattern. Abruptly interspersed with these undifferentiated areas were islands of cartilage and bone-like matrix with ossification, calcification, edema, focal abscess formation, and tumor necrosis involving <50% of the tumor (Fig. 1D–F). The tumor invaded through the colonic wall into the subserosal fibroadipose tissue and breached the mesothelium, with vascular-wall and neural invasion. No metastasis was found in the 33 regional lymph nodes. The tumor was Fédération Nationale des Centres de Lutte Contre le Cancer grade III, score 8.

Immunohistochemistry supported a malignant mesenchymal tumor and helped exclude its mimics. Tumor cells were positive for Vimentin (++), SATB2, TLE1 (++), CD99, INI-1 (+++), and Bcl-2 (+++), with focal CD117 positivity and weak STAT6 expression. CD34 highlighted the vascular network, and the Ki-67 index was ∼60%. The tumor was negative for CKPan (AE1/AE3), EMA, LCA/CD45, S-100, DOG-1, SMA, Desmin, p16, and IDH1. Masson staining was positive, and D-PAS was negative (Fig. 1G). Fluorescence *in situ* hybridization (FISH) showed *NCOA2* gene breakage/rearrangement in 85% of the counted tumor cells (Fig. 1H), whereas *SS18* (*SYT*) rearrangement was absent. Targeted next-generation sequencing (NGS) identified a *HEY1::NCOA2* fusion with an allele fraction of 29.7%. The integrated morphological, immunohistochemistry, FISH, and NGS findings established the diagnosis of primary colonic EMC.

Postoperatively, a multidisciplinary review incorporated the molecular pathology results, particularly the *NCOA2* rearrangement and *HEY1::NCOA2* fusion, which confirmed EMC and helped exclude key morphological mimics, including synovial sarcoma, Ewing sarcoma, and undifferentiated/dedifferentiated chondrosarcoma. Given the limited evidence for visceral EMC, surveillance and potential systemic therapy for high-risk or recurrent disease were discussed. After shared decision-making, the patient declined adjuvant therapy and chose close surveillance. She resumed a normal diet and remains under follow-up.

## Discussion

This case illustrates the diagnostic difficulty of primary colonic EMC. The previously reported colon-involving case arose from the retroperitoneum [2], whereas the present tumor formed an intramural colonic mass with mucosal ulceration and transmural invasion, supporting its primary colonic origin. Clinical manifestations are non-specific and depend on location and mass effect; in this case, fever, obstructive symptoms, abscess formation, and inflammatory exudation initially mimicked complicated colon cancer or inflammatory disease. Irregular calcifications in a hypermetabolic soft-tissue mass are useful radiological clues but are not pathognomonic [3, 4].

The differential diagnosis is broad. CKPan and EMA negativity argued against poorly differentiated carcinoma; SS18 negativity excluded synovial sarcoma. DOG-1 negativity and only focal CD117 expression did not support a gastrointestinal stromal tumor, and SMA/Desmin negativity argued against smooth-muscle differentiation. Although FLI1, ERG, and neuroendocrine markers were not available and should not be interpreted as negative, the classic biphasic morphology plus *NCOA2* rearrangement and *HEY1::NCOA2* fusion did not support Ewing sarcoma or a neuroendocrine neoplasm. Dedifferentiated chondrosarcoma was also considered; however, IDH1 negativity and the *HEY1::NCOA2* fusion favored EMC. *HEY1::NCOA2* is a highly characteristic molecular biomarker for mesenchymal chondrosarcoma [5–7].

Prognosis remains unfavorable, with late recurrence and distant metastasis possible even after complete resection; survival varies by age and tumor site [8]. Margin-negative resection remains the main potentially curative treatment [9]. Chemotherapy and radiotherapy have uncertain roles, although the cellular mesenchymal component may show partial sensitivity to anthracycline-containing or Ewing sarcoma-like regimens in selected patients [10].

In conclusion, this case highlights the need to consider EMC in calcified hypermetabolic gastrointestinal masses and to integrate histology, immunohistochemistry, FISH, and sequencing for diagnosis and postoperative management.

## Authors’ contributions

X.W. conceived and designed the project. H.J.L. and J.L.Z. collected the data. H.J.L. and J.L.Z. drafted the manuscript. All authors read and approved the final manuscript.
